# Neuromarketing Highlights in How Asperger Syndrome Youth Perceive Advertising

**DOI:** 10.3389/fpsyg.2020.02103

**Published:** 2020-10-07

**Authors:** Patricia Nuñez-Gomez, Anton Alvarez-Ruiz, Felix Ortega-Mohedano, Erika P. Alvarez-Flores

**Affiliations:** ^1^Department of Applied Communication Studies, Faculty of Media and Communication Science, Universidad Complutense de Madrid, Madrid, Spain; ^2^Department of Sociology and Communication, University of Salamanca, Salamanca, Spain; ^3^Unidad Académica Hermosillo, Universidad Estatal de Sonora, Hermosillo, Mexico

**Keywords:** Asperger syndrome, autism, ASD, neuromarketing, organizational communication, advertising perception

## Abstract

This study examines how advertising material and brands related to organizational communication are perceived by people with Asperger syndrome, a form of autism. The main objective of the study was to understand whether the perception of advertising differs between individuals with AS and a neurotypical population. Neuromarketing techniques were used to examine two key variables, attention and emotion, which were also measured by physiological and biometric variables. The results were compared with those of a control group from a neurotypical population; i.e., participants who had not been diagnosed with any type of developmental disorder. Commercial advertisements were the preferred material used in this research although social-themed advertisements were also included, some produced by commercial companies and others by institutional advertisers (NGOs and foundations). Qualitative techniques were also used to explain the observed phenomena. Data revealed significant differences between the two groups in their perception of advertising and organizational communication with respect to attention and emotion variables.

## Introduction

### Asperger Syndrome

Asperger syndrome denotes a form of what is more broadly known as Autism Spectrum Disorder (ASD). It is a disorder involving altered neurobiological brain development, characterized by restrained and repetitive behavior patterns and communication issues in several social contexts (American Psychiatric Association [APA], 2013). This means that people with Asperger’s develop a number of specific features. Even though ASD etiology is complex and still largely unknown, it probably results from the effects of genetic and environmental risk factors (Lai et al., 2014). It is worth noting that according to the new criteria set out in the Diagnostic and Statistical Manual of Mental Disorders or DSM-V (American Psychiatric Association [APA], 2013), Asperger syndrome is not recognized as a separate subtype of autism (as was the case with DSM-IV). Rather, it falls under the general category of ASD and is defined by DSM-V as an early appearance condition in children between 12 and 24 months of age (American Psychiatric Association [APA], 2013). Current data indicate that ASD prevalence in children is in the proportion of 1 in 59, being more common in men than in women (Levy et al., 2011; Jacquemont et al., 2014; Baio et al., 2018).

Broadly speaking, we can say that the “distinctive features” or general characteristics present in people affected by Asperger’s (hereinafter, AS) fall into three main areas, according to the Wing Triad (Wing, 1981):

Social area: limitations in establishing social relationships, lack of empathy, disproportionate emotional reactions, deficient interpretation of other people’s body language.

Communication area: verbal and non-verbal communication deficiencies, literality.

Psychomotor area: imperfect fine motor skills (American Psychiatric Association [APA], 2013).

Language can be idiosyncratic in people with ASD. This includes stereotypical language and unusual prosody, characterized by volume, tone, and also tonal and/or speech-rhythm abnormalities (Boucher, 2012; Matson, 2016). The occurrence of social communication problems in individuals with ASD is heterogeneous and often varies with age and the level of their cognitive ability (Rose et al., 2016). In most cases, people with ASD who show a better level of integration can present age-appropriate, or even advanced, communication abilities. However, even when their expressive language ability is intact, they often have a hard time developing social communication skills. These include effective communication of emotion and experience, engaging in reciprocal conversations, precise interpretation of figurative language (e.g., idiomsand metaphors), understanding contextual cues in conversation, and interpreting non-verbal cues in communication such as subtleties in body and facial language and voice intonation (Howlin, 2003; Kim et al., 2014; Peterson et al., 2019). This would have a direct impact on understanding and interpreting language in advertising material where, depending on the medium, these kinds of resources are widely employed to communicate adequately and achieve certain objectives.

To meet DSM-V diagnostic criteria for ASD, patients must show deficiencies in socio-emotional reciprocity, such as sharing interests and emotions, offering comfort to others, and/or initiating and responding to social interaction. Non-verbal communicative behaviors are affected in ASD, ranging from deficient integration of verbal and non-verbal behavior to anomalies regarding eye contact, use of facial expression and body language, to difficulties in interpreting or understanding non-verbal communication by other people. Lastly, people with ASD display significant deficits in social relationships, with the symptoms including a lack of social inhibition and motivation, and a failure to establish adequate friendships for their stage of development (Kasari and Rotheram-Fuller, 2007; American Psychiatric Association [APA], 2013).

People with ASD also show a deficit in ‘Theory of Mind’ or ToM, an aspect of social cognition described as the ability to identify and understand the thoughts, feelings, and intentions of others (Baron-Cohen et al., 2001; Peterson et al., 2019). ToM deficits cause social challenges, including difficulties in understanding and predicting the emotional states, behaviors, and outlook of other people, difficulties in inferring the motivations and intentions behind another person’s behavior and actions, and difficulties in distinguishing fact from fiction (Frith and Frith, 2006). In addition to these ToM deficits, people with ASD present significant deficiencies in the recognition and processing of emotions, as stated above. Such deficiencies remain for life (Boucher et al., 1998). In particular, it is believed that a persistent lack of attention to people’s faces results in subsequent impairment in recognizing facial expressions and emotions. The latter is a key component in non-verbal communication and successful social interaction (Baron-Cohen et al., 2001; Williams and Gray, 2013; Black et al., 2017). In addition to difficulties in recognizing emotions in others, people with ASD often struggle to identify emotions within themselves, along with contextual and physiological cues that facilitate emotional awareness (Hill et al., 2004; Rieffe et al., 2007; Kätsyri et al., 2008). It is worth noting that in recent decades most advertisements follow completely emotional codes. This complicates a literal reading of their messages, to the detriment of individuals with ASD. The central characteristics for ASD diagnosis, such as a restricted affection range and limited means of non-verbal communication, further alter emotional expression and responses to stress compared to the developing population (American Psychiatric Association [APA], 2013; Beker et al., 2018). All of these features must be taken into account when interpreting results and assessing message comprehension.

In addition to the basic criteria for ASD diagnosis, some characteristics and challenges commonly associated with areas of cognition and learning have been identified. These are likely to modify the way people with ASD experience and interpret their surroundings. For example, people with ASD tend to struggle with abstract reasoning, the ability to identify and formulate patterns, and dealing with concepts based on representative or abstract criteria. Studies have shown that people with ASD have a hard time carrying out classification tasks at a basic level when such a concept is used (Shulman et al., 1995). Many people with ASD exhibit learning issues, including difficulties in generalizing, a limited capacity to absorb new information, and slow processing speed (Peterson et al., 2019). Interestingly, however, despite deficiencies in abstract reasoning, many people with ASD show typically developed, or even advanced, visual-spatial processing skills, including the processing of visual patterns and object details (Kumar, 2013). Individuals with ASD are often visual learners, which means they understand or retain what they see more effectively than what they hear (Tissot and Evans, 2003). Moreover, though problems in the autobiographical memory area are ordinary, so are memory strengths in this population (American Psychiatric Association [APA], 2013; Meilleur et al., 2015). Some people with ASD can recall large amounts of information from limited stimuli, such as movie conversations or fragments of song lyrics they have heard only once. Thus, as will be explained later, they tend to watch their preferred audiovisual content more often.

This study aims to examine how AS subjects perceive advertising. It is important, therefore, to highlight the limitations of AS subjects with respect to social and communicative areas, particularly in language literality and the interpretative deficiency regarding other people’s body language. Abstract concepts and non-verbal communication are important features of advertising, but understanding such messages can be a challenge for subjects with AS.

### Advertising and Neuromarketing

In the last few years, neuroscience has allowed researchers to develop methodologies that complement older methods of studying the impact of marketing and publicity on consumers. Neuroscience studies the nervous system with particular emphasis on the brain. Consumer neuroscience is defined as “the application of neuroscience tools and theories to better understand the decision-making and brand consumption-comprehension processes” (Cacioppo, 2002; Plassmann et al., 2015, p. 427). It operates as an interdisciplinary field where neuroscience and consumer psychology intersect, with neuroscience supporting the study of decision-making processes.

Neuroscience has placed some theories under review, including stereotypical ideas and underlying social constructs that have underpinned consumer behavior (Azoulay and Kapferer, 2003). It has also been used to study the theory of mirror neurons using images obtained from functional Magnetic Resonance Imaging (fMRI) and by exposing individuals to video clips and facial expressions with positive, negative, and neutral emotions, demonstrating how certain areas of the brain related to these visual stimuli are activated. Such studies show from a neuroscientific perspective how the components of mirror neurons are triggered when looking at facial expressions with emotions (Bagozzi et al., 2012).

Brain activity can supply information unobtainable by traditional research methods such as focus groups, surveys, and interviews (Ariely and Berns, 2010). There is strong empirical evidence demonstrating a relationship between displayed stimuli and brain reactions, measured by electroencephalographic (EEG) techniques (Ohme et al., 2009). These commonly used methodologies in neuroscience can be extremely useful in studying the impact of communication such as publicity materials, giving rise to the new field of neuromarketing (Ramsoy, 2015).

Neuromarketing is a combination of neuroscience, psychology, and economics, involving the practical application of neuroscientific knowledge and techniques (often derived from consumer neuroscience) to obtain company-specific marketing information (Hubert and Kenning, 2008; Camerer and Yoon, 2015; Shaw and Bagozzi, 2017), and to measure the impact of commercial communication (Madan, 2010; Mañas-Viniegra et al., 2020). Neuromarketing helps researchers to overcome the limitations encountered in studying decision-making processes when consumers respond to brands. It also helps us to understand the processes that inform perceptions of advertising (Baron et al., 2017). The main reason for its use is the constraint displayed by individuals when consciously contributing information regarding their behavior through traditional techniques (Plassmann and Karmarkar, 2016; Mañas-Viniegra et al., 2019). Some authors stress the role of this science by highlighting the importance of measuring publicity data when combining attention and emotion (Gabrieli et al., 2015). This is especially relevant when complemented with surveys or other subsequent studies (Alalwan, 2018).

Neuromarketing has focused on measuring the efficiency of advertising campaigns and the behavioral psychology of consumers. The technique aids the study of attention and certain behaviors or attitudes, and understanding of how advertising and marketing campaigns impact the consumer’s mind (Lee et al., 2007; Ariely and Berns, 2010; Madan, 2010). It is a proven and valid method of measuring impact on the attention and emotions of a commercially targeted audience. Neuromarketing has provided companies with precise measurements that it is almost impossible to obtain by any other means. For example, when neuroscience is correctly applied and interpreted, the ability to predict the efficiency of certain commercials increases from 70 to 80% (Varan et al., 2015; Spence, 2019). This can enhance activities such as persuasive communication, where results have historically been hard to foresee and measure. With this in mind, neuromarketing was the primary tool used in this study, as it allows for the measurement of attention and emotion in AS individuals when responding to brands, and for a comparison with neurotypical subjects.

Many individual behaviors are not easy to observe using traditional research methods (Shaw and Bagozzi, 2017). They can be better measured with neuromarketing techniques, which provide researchers with objective, trustworthy, and less-prone-to-bias data (Camerer et al., 2005). In the case of advertising and organizational communication, an intricate decision-making process takes place through the neural system and functional circuits. Neurobiological components and cognitive and affective processes are dependent on the neural system, meaning that the simplified abstractions derived from areas of the brain and its neural circuits are especially useful. Such abstractions are the most essential elements in obtaining conclusions from the neurobiological markers that are fully substantiated by neuroscience.

Four main neural circuits have been established as important for consumer and decision-making neuroscience: (a) attention; (b) memory; (c) emotional processing; and (d) reward processing (Torreblanca et al., 2012; Shaw and Bagozzi, 2017). These variables are present when consumers are exposed to publicity. People are exposed to many stimuli, making it hard to discriminate and pay attention to all of them due to processing capacity. The exact process of attention is key in buying decisions. Different points of attention are correlated to various cerebral zones that can be observed and measured using neuroscientific techniques.

### Research Objectives

Few studies have been carried out with AS subjects in the communication area, especially in advertising. Studies have tended to focus on psychological and medical areas rather than on areas related to organizational communication or brand publicity. Due to its highly precise results and special characteristics for investigating audience responses to commercials, neuroscience is one of the most suitable methodologies for studying the reactions of people with AS. A mixed approach was adopted for this study, with neuroscience as the key technique. This was complemented by techniques used in social investigations to examine data obtained by neuroscience and find explanations for the observed behaviors.

The general aim of the study was to investigate the impact of commercial advertising on individuals with AS and to compare the results with those of neurotypical individuals. This was achieved by measuring their brand perception through emotion (EDR) and attention (EDL) variables. Additionally, the study had three specific objectives: (a) to investigate whether brands and companies are communicating appropriately with AS individuals; (b) to understand how a group of people diagnosed with AS assimilate, decode, and understand publicity; and (c) to understand how the language of advertising in its various forms influences AS and neurotypical individuals, as well as the meaning and use that advertising has for each group.

## Materials and Methods

### Participants

The sample comprised 32 subjects from the Madrid region (Comunidad de Madrid, Spain) distributed into two groups. Subjects were assigned to a group based on their neurological characteristics. The first group (the AS group) comprised 15 individuals diagnosed with Asperger’s (13 men and 2 women with a mean age of 22 years). The larger number of men was due to the higher occurrence of the syndrome in men than in women (Levy et al., 2011; Jacquemont et al., 2014; Baio et al., 2018). The second group (the control group) comprised 17 neurotypical individuals (11 men and 6 women with a mean age of 23 years).

A non-probability sample of opportunity was used to select the AS subjects, all of whom belonged to the Asperger’s Association Madrid. All members participated voluntarily and were informed about the research and its stages beforehand. Previous neuromarketing studies have determined that a sample size of between 15 and 50 subjects guarantees the validity of the research (Kerr-Gaffney et al., 2018; Zhang et al., 2018; Cuesta-Cambra et al., 2019; Mañas-Viniegra et al., 2020).

The research was conducted in compliance with the guidance of the Helsinki Declaration. The internal Ethics Committee of the department responsible for this investigation also approved this research. All of the 32 participants in the neuromarketing experiments and surveys were over 18 years of age. They all signed written authorization giving their consent to participate in the research and to the disclosure and transfer of data transfer. The Asperger’s Association Madrid approved the research method employed and supervised its development.

### Instruments and Materials

To determine the effectiveness of a stimulus with respect to its impact on attention and emotion, this study used instruments based on Sociograph technology which enable researchers to measure the unconscious reactions of a group of people exposed to commercials or audiovisual content. Sociograph is a company that specializes in peer-reviewed international neuromarketing studies (Aiger et al., 2013). Sociograph works with biometric technology developed by Salamanca University, Spain. The technology involves complex activation and control mechanisms that enable quantitative and simultaneous measurements of the mood of dozens of subjects at any given time, and consequently, the evaluation of emotional changes experienced when viewing communication items.

This technology operates by measuring electrodermal activity (EDA), one of the most significant and widely used indicators in neuroscience. It is a good indicator of attentional, emotional, and cognitive triggering processes in response to a collectively exposed stimulus (Dawson et al., 2007). It is worth noting that the unconscious interactions produced within a group when they view mass media and advertising indicate a common response to the emotional and cognitive stimuli. EDA is used extensively in the social sciences, not only for analyzing publicity and communication (Martínez et al., 2012; Tapia et al., 2016) but also for measuring anxiety or affectivity in psychiatry (Davidson, 2002).

In experiments that use Sociograph technology, subjects wear a bracelet on their right wrist. This technology broadcasts wirelessly to a central unit, where software processes and records real-time data from two biometric components: the level of attention (electrodermal level or EDL) and the level of emotion aroused by the material that is viewed (electrodermal response or EDR). The electrodermal activity (EDA) of up to 128 subjects can be registered simultaneously. Tonic activity, or EDL, is one of the most helpful variables to obtain an approximate measure of attention as it determines user agitation or alteration to the exposed stimulus. High levels indicate a greater predisposition to receive, analyze, and respond to the information (Tapia et al., 2016). Phasic activity, or EDR, is produced when a triggering stimulus causes a quick psychophysiological response in the subject. It is a good predictor of change after being exposed to a stimulus, making it one of the most commonly used indicators (Martínez et al., 2012). It does not distinguish between positive or negative emotions, only the physiological change provoked by the content; i.e., it indicates whether a stimulus has caused an emotional impact. Nevertheless, an increase in the subject’s emotional response can help to improve the effectiveness of a commercial (Lee and Hong, 2016). The higher the value, the stronger the emotion felt by the subject. Various studies have demonstrated a strong and linear relationship between emotion, personality, and affective state stimuli (Kucera et al., 2004; Crider, 2008). The two measures obtained through this system (EDL and EDR) correspond exactly with the two main previously noted social problems of AS individuals: lack of continuous attention and a lack of emotional projection.

For the advertising material in the test, a representative sample of commercials was chosen from broadcast television due to its comprehensive depiction of the companies and the brand values that are advertised. Additionally, thanks to its audiovisual content, it offers a more compelling sensorial experience compared with advertisements from other media. The average duration of the commercials was between 20 and 60 s to avoid people with AS experiencing boredom. A total of 33 commercials from companies and brands in different commercial categories were used. These also included some commercials from social and public interest entities.

An important factor was that brands exert a rhetorical function as signals, making commercials more complex by elevating their level of abstraction (McQuarrie and Glen, 1996; Malkewitz et al., 2003; Phillips and McQuarrie, 2003). In such cases, the product or brand is associated with values that awaken emotions in the audience, meaning they identify with the brand and become attached to it (Ducoffe, 1996; Roberts, 2004; Taylor et al., 2011; Lee and Hong, 2016). Taking this into account, the study focused on communication and classified commercials into three groups based on how easy it was to understand them, detailed in Table 1.

**TABLE 1:** Characteristics and distribution of commercials based on complexity level.

Commercial complexity	Characteristics	Number and reel duration*
Low	A simple, easy-to-understand narrative is offered. Content is presented literally using common situations. The viewer is not required to complete the message or decode rhetorical figures. Brand is easily identifiable and relatable to the contents.	10 commercials; 3 min 7 s
Medium	Messages are harder to comprehend. Rhetorical figures are frequent, mainly metaphor and irony. Brands tend to be easily identifiable, but their relationship with the content is often indirect or subtle.	12 commercials; 12 min 9 s
High	Content is only slightly literal, often ambiguous and ill-defined. The use of hard-to-interpret devices such as metaphor is widespread, along with the superimposition of several simultaneous rhetorical figures. This complicates message decoding, demanding much attention from the viewer, and an imaginative reinterpretation of the content. Brands are usually not easy to identify, and their relationship to the product is not evident.	11 commercials; 15 min 12 s

**^∗^Though the number of commercials is similar in each category, the duration of the reel was not, since medium and high complexity commercials are longer on average*.*

In order to identify the types of commercials used in the experiment, we set up a dynamic focus group with a reduced format. This comprised a group moderator and a few AS subjects to avoid them being inhibited. The usefulness of focus groups with a reduced number of participants has been validated in other scientific literature (Ibáñez, 1979, p. 273). In terms of the focus group dynamics, it was difficult to keep the AS subjects interested and explore topics in-depth, and we decided to carry out in-depth interviews with other AS individuals a day after the group session to obtain more focused opinions.

To qualify and explain the results from the Sociograph viewings, Alalwan’s (2018) recommendation was taken into account, and we asked participants to complete either a short survey or written test. The duration was kept short to allow for the attention needs of AS subjects. The survey or test was related to three specific commercials that were considered particularly representative of each of the complexity categories. The test asked the participants whether they liked the commercial and to explain why. Their understanding of the product and the company image was then analyzed. The test for the neurotypical group was identical to that for the AS group.

### Procedure

Two sessions were carried out, one for the AS group and another for the control group. Both were held on April 10, 2019, at the premises of the Asperger’s Association Madrid, a place that was familiar to AS participants. The 33 commercials were mounted on a reel, randomly mixing the three categories of complexity, with a total run time of 30 min and 28 s. The videos were shown in full and in the same order in both sessions. The first 7 min of the commercials were not counted, because previous studies carried out with Sociograph technology have shown that measurements at the start are not reliable due to the initial excitement created by the situation of the experiment. This effect gradually decreases and disappears completely after a few minutes. The written surveys were completed immediately after the neuromarketing sessions.

### Data Analysis

Sociograph technology allows for the mathematical analysis and processing of data in a set timeline with specific algorithms. Data are obtained as coefficients, which can be translated into a graph. This application can measure EDL and EDR parameters with a frequency of 36 inputs per second. For this type of study, 1 input per second is more than enough. The application calculates this measure by averaging those 36 inputs per second (Tapia et al., 2016).

Tonic activity (EDL) is measured using the sum of all participants’ electrodermal resistance in kiloohms (KΩ). The less resistance obtained from the exposed subject, the higher the attention level. Higher resistance is associated with a lower degree of attention. To facilitate interpretation, EDL values were inverted in the Excel graphic representation. An increased EDL on the time graph represents an increased attention level, whereas a decreased EDL on the time graph represents a decreased attention level. The research results were grouped into statistical data to ensure participant anonymity, and prevent the identification of the respondents.

## Results

The results are based primarily on data obtained from Sociograph technology. Where necessary, this incorporated data obtained from the surveys, focus groups, and in-depth interviews.

### Similarities and Differences in Attention and Emotional Responses

The variety of advertisements in the tests produced a large amount of data. This allowed a detailed study of each commercial. However, a summary of the main observations was deemed to be of greater interest. In particular, we compare the responses of AS and control subjects and attempt an explanation of notable differences. As expected, differences between the AS and control participants were very significant, both in how a commercial was understood and in the attention and emotion levels of the subjects. As discussed below, the evolution of the variables for some commercials was inverted in the two groups.

The first observation was that the EDL graph (attention level) and EDR graph (emotion level) were not interdependent for either group (AS and controls). For example, at a time when attention decreased in EDL, a high peak of emotional response was detected in EDR. However, both variables crossed throughout each commercial and we were able to determine whether the varying levels of attention and emotion represented an effective commercial (i.e., one that attracts attention and creates expectation based on the sales proposal or the values expressed by the brand).

**FIGURE 1:**
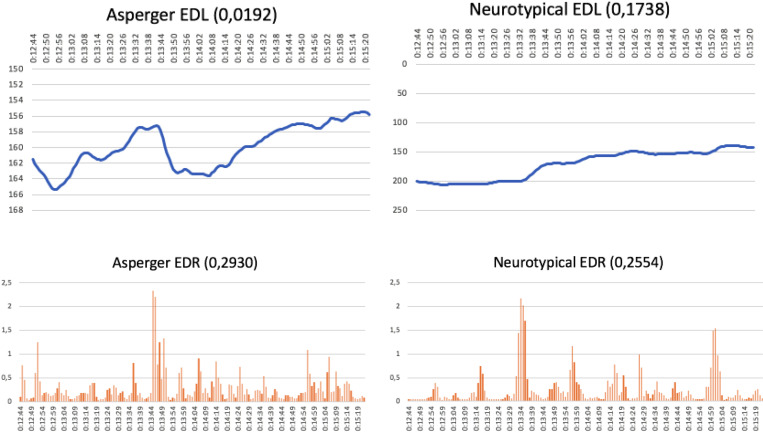
Ikea commercial: “The Other Letter” (running time: 12:44–15:20 min) (https://www.youtube.com/watch?v=fQ2kFqq6Ldo).

An important result concerns the perception of the two groups. The interest level and emotional response tended to be similar for both groups when a descriptive commercial told an easy-to-follow, chronological story. In this case, AS subjects seemed to have no problem understanding fast montages or accelerated images. They found it more difficult to accept long pauses between moments with narrative tension, slow rhythms, or commercials longer than a minute. For instance, take the EDL graphs representing the interest level for Ikea’s “The Other Letter” commercial (Figure 1). They show a similar development for the AS group (top left) and the control group (top right). Even though the intent of this commercial makes it highly complex, its story is chronological and maintains a natural, continuous, easy-to-follow rhythm. This would explain why attention levels follow a similar pattern in both groups, with differences indicated by the pronounced “saw teeth” in the AS graph where the control graph shows a smoother evolution.

**FIGURE 2:**
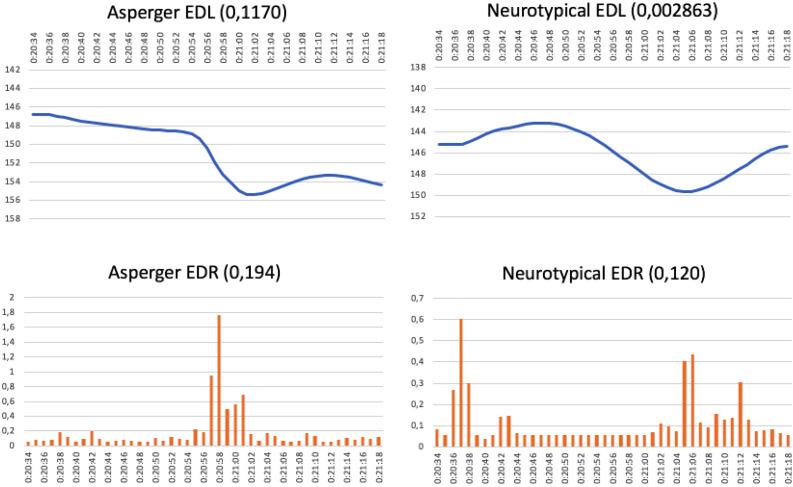
McDonald’s commercial: “Great Menu” (running time 20:34–21:18 min) (https://www.youtube.com/watch?v=VVn1R0rMVLI).

Figure 1 also shows the EDR graphs for emotional response. Here, both audiences reacted at almost the same moments in the commercial, although the control graph shows a greater number of emotional peaks. This also happened with the McDonald’s “Great Menu” commercial, where the EDL graphs for attention follow similar patterns in the two audiences (Figure 2). This is ascribed to a very short (10 s), low complexity, easy-to-follow story. In this case, however, the EDR graphs for emotional response are very different and reach peaks at completely different times for the two groups, with controls reacting more often (at 20:37, 21:06, and 21:12) than the AS group, whose only emotional peak was at 20:58.

When plot intricacies appear or the story is hard to follow (e.g., images requiring conscientious reading, non-chronological montages), the initial interest among AS subjects increases rapidly. However, if the situation lengthens with no apparent explanation, or if it becomes increasingly complicated, attention to the message decays. By contrast, the neurotypical audience responded with greater caution to unexpected or complicated scenarios. Their attention was not triggered so quickly and grew with ups and downs. When the plot was resolved in a clever or sentimental manner, both attention and emotion graphs rose. AS subjects show discontinuities and jumps in attention that could be due to being more instantaneous, and their reactions dependent on the specific stimuli of each moment and expressed spontaneously. In contrast, the attention graphs for neurotypicals show a progressive evolution because they follow a gradual process and, most likely, manage their attention with greater self-control. This could explain the graphs for some commercials, where a comparison of the EDR (emotional response) and EDL (attentional response) for the AS group and neurotypicals shows that the graphs seem to be opposite, even mirror-like.

**FIGURE 3:**
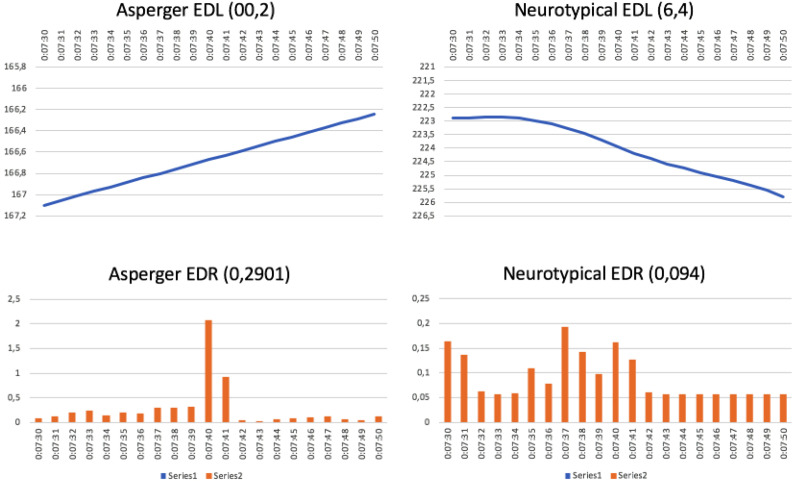
Vileda kitchen cloth commercial: “Magical” (running time: 7:30–7:50 min) (https://www.youtube.com/watch?v=TzqdOVRbgoI).

Typically, in these cases, interest and emotion in the neurotypical audience gradually rise in a sawtooth fashion, following storylines that are set out to reach a crescendo. However, the AS audience can react to the same stimuli with the almost opposite response. There are commercials where attention among neurotypicals decreases from the first few scenes due to a predictable development, whereas attention among the AS group increases because the plot simplicity allows them to follow it with little difficulty. This can be seen in the Vileda kitchen cloth commercial (Figure 3), where AS attention gradually increases (top left), whereas the opposite happens for the neurotypical group (top right). As for the emotion curve, there are significant differences between the groups. The AS graph (bottom left) shows a flat and low curve with pronounced peaks at 07:40 and 07:41, which correspond to increases in the neurotypical graph. The AS graph suddenly drops and emotional response almost disappears until the end. Contrary to this, the neurotypical curve (bottom right) is much more active, with gradual ups and downs, and six peaks (at 07:30 and 07:31; 07:37, and 07:38; 07:40 and 07:41).

### Other Points of Interest

As mentioned above, the graphs for the AS group show bursts of emotion as the images, sounds, and situations seem to excite their perception, but their attention decreases more quickly. AS subjects show an initial interest in situations that are difficult to understand, possibly because they expect to find an element of meaning. However, if that does not happen right away, attention and emotion levels fall quickly. The levels of attention and emotion can be recovered by the appearance of a new element if it creates the expectation of an explanation. During the focus group and in-depth interviews, some AS subjects said they had problems understanding puns and double-entendres. This difficulty led to an increase in interest, but this attentiveness decays rapidly when non-comprehension continues for a long time.

The appearance of an isolated element such as a big picture, a flashy scene, or a special sound, can trigger a sudden and brief rise in the emotional variable for the AS group. This does not happen with a neurotypical audience, because changes in their attention and emotions are more subtle, with a smoother transition. Their attention grows suddenly in commercials where the viewer gradually discovers a hidden meaning that was not apparent at the beginning, or when an unexpected turn of events occurs, or when a new interpretation of the message is possible. Such increases in attention do not happen with AS subjects, probably because they do not have an immediate grasp of the intentions. Similarly, lengthy ambiguous stories or scenarios that arouse interest in neurotypicals do not have the same effect for AS subjects. This is probably because the former expect a denouement, whereas the latter are unable to obtain a clear message and their progress is therefore prevented.

The presence of celebrities is a technique employed by the advertising industry, as it has been shown to increase attention and memory rates, both in commercial brands and social advertisements. Famous people were included in our reel of commercials and the effects corroborated the positive reaction of a neurotypical audience. However, this type of commercial did not seem to have a significant effect on the AS audience whose attention was not roused, neither were emotional responses generated.

### Three Levels of Complexity

When considering the complexity of the plot of the commercials, neurotypicals liked complicated stories as long as they could be understood. This was apparent for every commercial. By contrast, AS subject comprehension was inversely proportional to the level of plot complexity. However, that did not appear to frustrate or discourage them (which could dangerously undermine the advertised brand or company) but generated a lack of interest in the advertisement. As previously stated, three of the commercials tested were subjected to deeper research, based on the three levels of complexity found in current advertising. The following is an analysis of each of the three commercials, from the lowest to the highest level of complexity. The analysis draws on the knowledge obtained from focus groups and in-depth interviews.

#### Low Complexity Commercial

**FIGURE 4:**
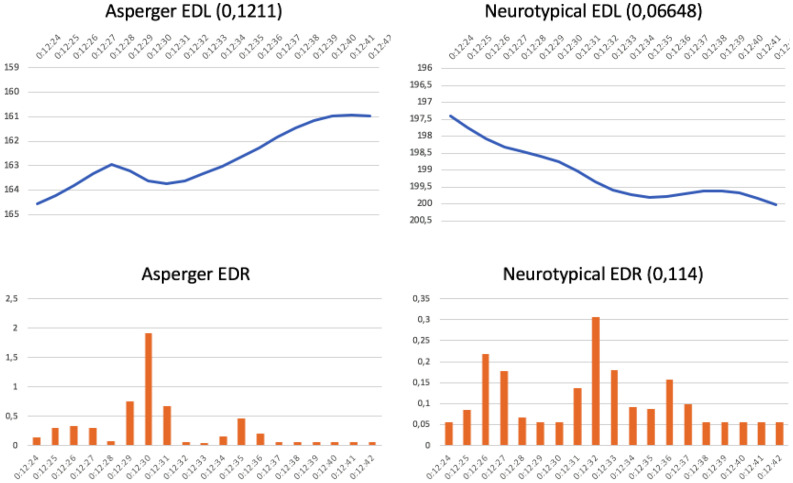
Dyson vacuum cleaner commercial: “Cordless” (running time 12:24–12:42 min) (https://www.youtube.com/watch?v=twPbou1ix2o).

The first commercial comprises a product demonstration (Dyson vacuum cleaner commercial, “Cordless”^1^). In this commercial for Dyson, a housewife is cleaning her house with ease thanks to the features of a new vacuum cleaner, which does not need a power cord. In this case, the survey results were even between the two groups. Regarding comprehension, significant proportions of both groups understood the message: 82.3% of neurotypical and 64.7% of AS subjects. Both also agreed that selling the product was the sole message (70.5% of neurotypicals and 76.4% of AS subjects). The control group did not like the commercial due to its simplicity and obviousness (47% did not like it at all and 35.3% liked it a little). In contrast, 41.17% of the AS audience liked it a lot. The reason, according to their statements, was that “they understood it perfectly.” This group highlighted in their comments that they liked it because it was “simple,” “clear,” “useful,” and “well-explained.” Neuromarketing graphs (Figure 4) confirm these opinions. Interest rises in the AS group (top left) reached a significant emotion peak at 12:30 (bottom left), whereas neurotypical interest progressively fell as they watched the commercial (top right) due to predictability, despite having some minor peaks of emotion (bottom right).

#### Medium Complexity Commercial

**FIGURE 5:**
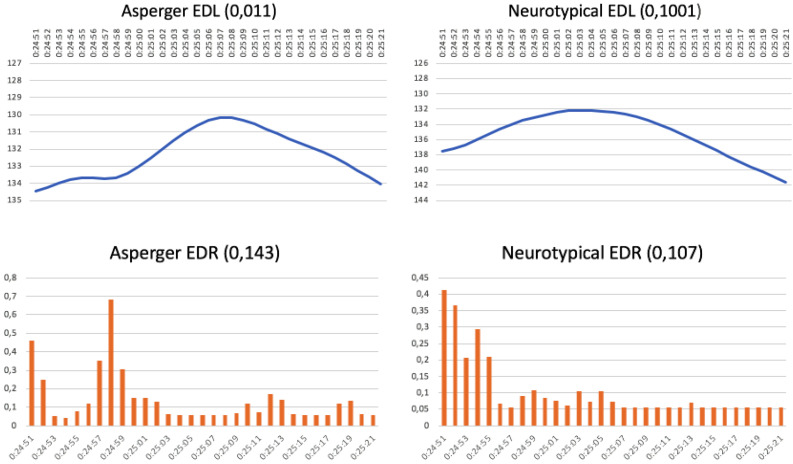
Pizzas Casa Tarradellas commercial: “Pirates” (running time 24:51–25:21 min) (https://www.youtube.com/watch?v=HyCEUlsMTmA).

The second commercial showed a family story (Pizzas Casa Tarradellas commercial “Pirates”^2^). In this commercial, a boy dressed up as a pirate does not want to bathe before dinner because, he says, “pirates don’t bathe.” His mother manages to convince him to take a bath by offering him a pizza, which the entire family enjoys together in the last scene. Despite its simple content, the commercial is a little difficult to follow due to several ellipses in the narrative timeline that the viewer is expected to fill. Here we begin to find significant differences between the two groups. While 76.5% of the neurotypicals understood the advertisement perfectly, only 40% of the AS subjects managed to do so, with 33.3% understanding it only a little. In general, the commercial was enjoyed by both groups – neurotypicals slightly more so – due to its emotivity, the familiar situation, and the deliberate use of humor. These qualities compensate for the AS group’s difficulties in comprehension, which was also indicated by feedback from the surveys and in-depth interviews. This is reflected in the neuromarketing graphs shown in Figure 5, which look very similar in terms of interest evolution. As usual, the curve is more pronounced for the AS group.

#### High Complexity Commercial

The third commercial depicted a highly complex story summarized in a few takes (ONCE commercial “Christmas Lottery”^3^). This example depicted a family gathering a few days before Christmas. One of them is about to hand out coupons to all of them for a lottery with a very high cash prize (a very common situation in Spain at Christmas). One by one, the family members explain what they plan to do should they win the prize. They all want to go far away to enjoy the money, meaning that the family would be separated and the person with the coupons decides not to distribute them, preserving family unity. The commercial is difficult to understand because of the underlying meanings, and also because of the complex situation where the world of desires (“what I would do if I were a millionaire”) and the real world are compared. Of the neurotypical group, 100% claimed to understand the story. Undoubtedly and despite its complicated plot, the commercial was interesting and created enough intrigue for viewers to follow the story. Of the AS subjects, 60% thought they understood the commercial but, unlike the two commercials above, an analysis of their explanations revealed that they misunderstood the story. They made several incorrect assessments of the situation; e.g., they assumed that the family had already won the lottery rather than reading it as a mere possibility. Only 6.6% of the AS subjects stated explicitly that they “did not get it at all.”

**FIGURE 6:**
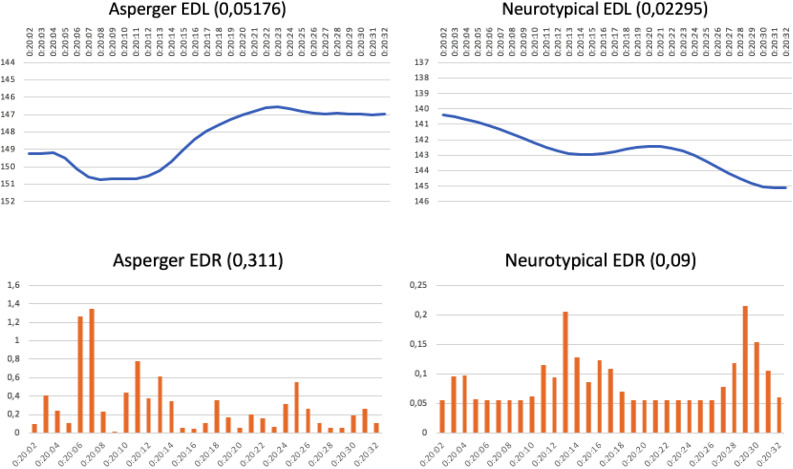
ONCE commercial: “Christmas Lottery” (running time 20:02–20:32 min) (https://www.youtube.com/watch?v=E70b9EK9Chg).

The neurotypical group labeled the commercial as attractive and this particular advertising campaign was a great success. The AS group, however, considered the commercial to be “a little attractive” (53.8%) or “not at all attractive” (26.6%). The neuromarketing graphs shown in Figure 6 confirm these effects. In terms of the evolution of attention, neurotypical interest decreases as they predict the outcome of the commercial, whereas interest among the AS subjects increased as the ambiguity of the situation builds an expectation that there will soon be an explanatory element in the plot. However, as their expectation is not fulfilled, AS subjects feel disappointed. The emotion graph verifies that the level of emotional response created in the neurotypicals maintains a high average throughout the entire commercial, as well as showing two peaks (at 20:13 and 20:29), one nearly at the end. As usual, the AS graph presents many “saw teeth,” when compared to the control group and emotional engagement is much less intense throughout the commercial.

Analysis of the three levels of complexity in current advertising indicated that storyline complexity, which is a common approach in recent commercials, widens the ever-increasing gap between the general public and AS individuals. While the former prefer smart and complex stories (as long as they are interesting), the latter feel confused or believe they have understood the message correctly when they have misunderstood it, which leads to frustration and disappointment. This happens even when their interest has gradually increased throughout the viewing. This is a deceptive effect, as the AS subject has not enjoyed the commercial, its message has not been effectively conveyed, and it does not create positive feelings toward the brand or company.

## Discussion

One of the greatest challenges our society currently faces is developing activities that respect the diverse needs of different people. This becomes particularly relevant when it comes to communication and more specifically, advertising. The latter sends out messages, transmits values, and reflects stereotypes, routines, and lifestyles that influence society, with negative or positive effects on citizens. This study aimed to determine whether the perception of advertising differs between subjects with AS and a neurotypical population, using different types of commercials with varying degrees of complexity.

The level of complexity in commercials was shown to affect both groups but in different ways. In the AS group, low-complexity commercials were among the most prominent in the EDL attention graphs: almost all of them were in the upper half of the score range. On the other hand, EDR emotion graphs rank medium and high-complexity commercials highly. This appears to indicate that, although they are not clearly understood by AS individuals, rhetorical stories have a greater capacity to focus their feelings. Low-complexity commercials were situated in the lower half of the EDR rankings.

By contrast, neurotypicals showed a more varied distribution in the EDL and EDR graphs in terms of complexity level. Interest in stories is most likely more determinant in this group than the level of complexity. This difference indicates that for the AS audience, comprehension difficulty is the main variable that determines whether a commercial is attractive or awakens feelings, and this variable greatly determines the importance of other elements in the commercial (Howlin, 2003; Kim et al., 2014; Peterson et al., 2019).

These observations suggest that the impact of advertising communication on AS individuals reinforces and expands the problems already faced by this group. AS subjects have difficulties relating to others, which also affects their relationship with brands and company communications. As explained in the introduction, these difficulties can affect their understanding of their surroundings, restrict their social lives, determine their level of communication with others, and capacity for empathy (American Psychiatric Association [APA], 2013).

Concerning sale expectations and generating brand and company content, it can be concluded that the most influential commercials for AS individuals are those with clear and direct messages. These tend to discuss product characteristics, the advantages of its use, brand values, and are related to the brand and the company. Linear and chronological storylines that advance progressively and without great leaps are also more effective. It was observed that incorporating some unexpected scenes that do not fit into the linear narrative, but which are quickly explained, generates immediate attention and peaks in emotion in an AS audience. On the other hand, the difficulty of interpreting facial expressions can make a commercial more incomprehensible (Gepner et al., 2001).

This research has also highlighted that some advertising has an opposite effect on the general public compared to AS individuals. For the general public, there is a directly proportional relationship between plot complexity and pleasure, provided the situation is thoroughly resolved. In other words, a neurotypical audience enjoys complicated commercials, which are followed attentively and excitedly, as long as they provide sufficient elements for comprehension (Wing, 1981; Rose et al., 2016). Contrary to this, in the AS audience, we observed an inverse relationship between the difficulties of understanding and pleasure. The greater the difficulty in understanding, the worse the final impression left by the commercial (Beker et al., 2018). Considering that people with Asperger’s tend to watch the audiovisual products they enjoy over and over (Shane and Ducoff, 2008), the content emitted by companies should be assessed, and the underlying intention to persuade should be made clearer. This research has shown that advertising can be used to teach emotions and feelings to these audiences (Norman et al., 2001; Corbett, 2003; LeBlanc et al., 2003) and, given the importance of these issues, further studies on this topic should be conducted.

## Data Availability Statement

The raw data supporting the conclusions of this article will be made available by the authors, without undue reservation, to any qualified researcher.

## Ethics Statement

The studies involving human participants were reviewed and approved by Asperger’s Association Madrid and the ethics committee of the Department of Applied Communication Studies in Complutense University of Madrid. The patients/participants provided their written informed consent to participate in this study. Written informed consent was obtained from the individual(s), and minor(s)’ legal guardian/next of kin, for the publication of any potentially identifiable images or data included in this article.

## Author Contributions

PN-G and AA-R were involved in the conceptualization of the project and acquisition of data and analysis. PN-G, AA-R, FO-M, and EA-F were involved in the interpretation of the data. All authors were involved in drafting and revising the work for intellectual content and approved the manuscript for publication.

## Conflict of Interest

The authors declare that the research was conducted in the absence of any commercial or financial relationships that could be construed as a potential conflict of interest.

## Footnotes

1 https://www.youtube.com/watch?v=twPbou1ix2o

2 https://www.youtube.com/watch?v=HyCEUlsMTmA

3 https://www.youtube.com/watch?v=E70b9EK9Chg
