# Supplementary material for: Neuromarketing Highlights in How Asperger Syndrome Youth Perceive Advertising
Source: Front Psychol. 2020 Oct 7;11:2103. doi: 10.3389/fpsyg.2020.02103 (PMC7575727; doi:10.3389/fpsyg.2020.02103)
Supplement: Supplementary file 1 [file Data_Sheet_1.ZIP › Supplementary Material Presentation/_Table 1.docx]

**Table 1**. Characteristics and distribution of commercials based on complexity level

| Commercial complexity | Characteristics | Number and reel duration* |
| --- | --- | --- |
| Low | A simple, easy-to-understand narrative is offered. Content is presented literally using common situations. The viewer is not required to complete the message or decode rhetorical figures. Brand is easily identifiable and relatable to the contents. | 10 commercials; 3min. 7sec. |
| Medium | Messages are harder to comprehend. Rhetorical figures are frequent, mainly metaphor and irony. Brands tend to be easily identifiable, but their relationship with the content is often indirect or subtle. | 12 commercials; 12min. 9sec. |
| High | Content is only slightly literal, often ambiguous and ill-defined. The use of hard-to-interpret devices such as metaphor is widespread, along with the superimposition of several simultaneous rhetorical figures. This complicates message decoding, demanding much attention from the viewer, and an imaginative reinterpretation of the content. Brands are usually not easy to identify, and their relationship to the product is not evident. | 11 commercials; 15min. 12sec. |

*Though the number of commercials is similar in each category, the duration of the reel is not since medium and high complexity commercials are longer on average.
